# Multimodal imaging of structural damage and inflammation in psoriatic arthritis: a comparison of DMARD-naive and DMARD-failure patients

**DOI:** 10.1093/rheumatology/keae450

**Published:** 2024-08-17

**Authors:** Nağme Ö Renkli, Nienke J Kleinrensink, Julia Spierings, Simon Mastbergen, Harald E Vonkeman, Shasti C Mooij, Lydia G Schipper, Amin Herman, Iris ten Katen, Frank J Nap, Marjolein E Hol, Pim A de Jong, Mylène P Jansen, Wouter Foppen, the TOFA-PREDICT Author Group, Kavish J Bhansing, Sandra T A van Bijnen, Radjesh J Bisoendial, Antoaneta C Comarniceanu, Lenny Geurts-van Bon, Z Nazira Jahangier, Tim L T A Jansen, Marc R Kok, Arno W R van Kuijk, Emmerik F A Leijten, Astrid M van Tubergen, Simone A Vreugdenhil, Siska Wijngaarden

**Affiliations:** Department of Rheumatology and Clinical Immunology, UMC Utrecht, Utrecht University, Utrecht, The Netherlands; Department of Rheumatology and Clinical Immunology, UMC Utrecht, Utrecht University, Utrecht, The Netherlands; Department of Radiology and Nuclear Medicine, UMC Utrecht, Utrecht University, Utrecht, The Netherlands; Department of Rheumatology and Clinical Immunology, UMC Utrecht, Utrecht University, Utrecht, The Netherlands; Department of Rheumatology and Clinical Immunology, UMC Utrecht, Utrecht University, Utrecht, The Netherlands; Department of Rheumatology, Medisch Spectrum Twente, Enschede, The Netherlands; Department of Psychology, Health and Technology, University of Twente, Enschede, The Netherlands; Department of Rheumatology, Medisch Spectrum Twente, Enschede, The Netherlands; Department of Rheumatology, Elisabeth- TweeSteden Hospital, Tilburg, The Netherlands; Department of Rheumatology, Antonius Hospital, Utrecht, The Netherlands; Department of Radiology and Nuclear Medicine, UMC Utrecht, Utrecht University, Utrecht, The Netherlands; Department of Radiology and Nuclear Medicine, UMC Utrecht, Utrecht University, Utrecht, The Netherlands; Department of Radiology and Nuclear Medicine, UMC Utrecht, Utrecht University, Utrecht, The Netherlands; Department of Radiology and Nuclear Medicine, UMC Utrecht, Utrecht University, Utrecht, The Netherlands; Department of Rheumatology and Clinical Immunology, UMC Utrecht, Utrecht University, Utrecht, The Netherlands; Department of Radiology and Nuclear Medicine, UMC Utrecht, Utrecht University, Utrecht, The Netherlands

**Keywords:** psoriatic arthritis, PsA, imaging, MRI, conventional radiographs, PET/CT, HEMRIS

## Abstract

**Objectives:**

To compare inflammatory and structural differences in active PsA between DMARD-naive and DMARD-failure patients using diverse imaging approaches for future analyses. Additionally, to explore the influence of patient characteristics (clinical and demographic variables) on imaging findings.

**Methods:**

Of the 80 patients included from the first cohort of the ongoing multicentre TOFA-PREDICT trial, 40 were DMARD-naive and 40 were DMARD-failure (csDMARD failure; one prior bDMARD excluding etanercept was allowed), all meeting classification criteria for PsA with a minimum disease duration of eight weeks. Baseline conventional radiographs of hands and feet, MRIs of both ankles, and whole-body [^18^F]-fluorodeoxyglucose PET/CT (^18^F-FDG PET/CT) were evaluated for inflammatory and structural imaging parameters, including Sharp-van der Heijde (SHS), Heel Enthesitis Magnetic Resonance Imaging Scoring System (HEMRIS) and Deauville synovitis scoring. Differences between groups and the influence of patient characteristics were examined with multiple linear regression.

**Results:**

At baseline, patient characteristics were similar between groups. Imaging parameters showed limited inflammation and structural damage. Inflammatory imaging parameters were not significantly different (*P* > 0.200). Among structural parameters, only HEMRIS Achilles tendon structural damage was significantly different (*P* = 0.024, R^2^ = 0.071) and SHS Joint Space Narrowing was not statistically significant (*P* = 0.050, R^2^ = 0.048) with higher values for both in DMARD failures. After correction of patient characteristics, these differences in imaging disappeared (both *P* > 0.600).

**Conclusion:**

At baseline, PsA patient groups were comparable concerning structural and inflammatory imaging parameters, especially after correcting for patient characteristics. Thus, DMARD-naive and DMARD-failure patient groups may be combined in future PsA progression and treatment decision studies.

**Trial registration:**

www.clinicaltrialsregister.eu. EudraCT: 2017–003900-28.

Rheumatology key messages

- DMARD-naive and DMARD-failure PsA patients displayed comparable inflammation, and structural damage on imaging.
- In our study, failing a DMARD was not associated with worsened imaging findings.
- DMARD-naive and DMARD-failure patients may be combined (after correction of patient characteristics) for future analyses.

## Introduction

PsA is a complex, chronic inflammatory and heterogeneous musculoskeletal disease which may arise in up to 30% of psoriasis (PsO) patients [1, 2]. The heterogeneity of PsA leads to challenges in identifying an effective DMARD for an individual patient [3]. Comparing different patient profiles would improve the understanding of underlying differences that might be contributing to varying outcomes and optimizing the treatment response. However, to this date, limited research is available comparing patients who never used DMARD *vs* patients who previously used DMARD using diverse radiographic manifestation of PsA. Thus, more insight into the structural and inflammatory manifestation of PsA using various imaging approaches in these different patient profiles is needed.

Different medical imaging techniques can help us investigate the heterogeneous manifestation of PsA by examining a range of inflammatory and structural outcomes. Three imaging techniques that can be used for this examination are conventional radiograph, MRI and ^18^F-FDG PET/CT. Conventional radiographs are valuable for assessing structural damages, particularly in the frequently involved joints of the hand and feet [4]. The Sharp–van der Heijde score (SHS) adapted for PsA is a well-established method for conventional radiographs to score the erosion and joint space narrowing in the hands and feet [5].

MRI is a frequently used technique to assess both inflammatory and structural damage [6]. Applying the recently developed scores Heel Enthesitis Magnetic Resonance Imaging Scoring System (HEMRIS) and Psoriatic Arthritis Magnetic Resonance Imaging Scoring System (PsAMRIS) by OMERACT group [7, 8] on MRI scans provide ways to capture the different aspects of the disease such as bone erosion and inflammation.^18^F-FDG PET/CT is another valuable technique to detect inflammatory manifestation of PsA [9]. It can be used to evaluate synovitis using the most commonly affected large synovial joints such as the shoulder, knee and ankle [4, 10]. In addition, this technique allows the assessment of systemic inflammation, by evaluation of aortic vascular inflammation [11, 12].

In the literature on PsA, various imaging techniques were used to analyse the disease characteristics of PsA. However, no research combined the described imaging techniques and scores to comprehensively capture the heterogenic manifestation of PsA. Additionally, to the best of our knowledge, no previous studies compared two distinctive patient groups: DMARD-naive [those who have never used conventional synthetic DMARD (csDMARD)] and DMARD-failure (non-responders to previous csDMARD treatment) using both structural and inflammatory imaging assessment methods. Therefore, this study aimed to characterize the impact of PsA in two different patient groups with active PsA, namely DMARD-naive and DMARD-failure patients as an exploratory analysis to identify potential underlying trends and associations for future studies. Thus, the objective of these analyses was to explore potential differences in inflammatory and structural imaging parameters between DMARD-naive and DMARD-failure patients at baseline and to evaluate the influence of patient characteristics (clinical and demographic variables) on the observed differences in inflammatory and structural imaging parameters for future PsA management studies.

## Methods

### Study design and patients

Patients with PsA and active disease were included in the TOFA-PREDICT multicentre trial that studies therapy response prediction in PsA (EudraCT 2017–003900-28). This ongoing trial is conducted in The Netherlands and coordinated by the University Medical Center Utrecht. Participants in this study fulfilled the following criteria: meeting the classification criteria for psoriatic arthritis (CASPAR) [13], aged 18–75 years, a disease duration of a minimum of eight weeks, and evidence of active peripheral arthritis (≥2 swollen joints and ≥2 tender joints). Details about the inclusion and exclusion criteria and study design can be found in the previously published study design paper [14].

While in the TOFA-PREDICT trial, patients receive treatment and are followed over time. The current study is a cross-sectional evaluation of patients with active PsA at baseline. The original TOFA-PREDICT study was appropriately powered for sample size but no formal power calculation was made for this sub-study. In total, the TOFA-PREDICT trial will include two cohorts of 80 PsA patients with active disease. For this study, the first cohort of 80 patients were used since the inclusion for this cohort was completed, whereas in the second cohort, inclusion is still ongoing. Among these 80 patients, 40 were DMARD-naive patients who had previously not used any DMARDs (conventional or targeted synthetic, or biologic). The remaining 40 were DMARD-failure patients who did not respond sufficiently to previous csDMARD treatment (prior use of one bDMARD excluding etanercept was allowed). Baseline patient characteristics and the following baseline imaging studies were analysed from these patients: conventional radiographs of the hands and feet, MRI scans of both ankles and whole-body ^18^F-FDG PET/CT. All the patients included in this study provided written consent and the study was approved by the Medical Research Ethics Committee in Utrecht, Netherlands (MREC reference number: NL63439.041.17).

### Clinical assessments

Baseline patient characteristics for PsA patients were as follows: age, gender, BMI, smoking status, time since diagnosis of psoriatic arthritis (years), time since diagnosis of psoriasis (years), psoriasis area and severity index (PASI), HAQ [15]. Laboratory evaluation included CRP. Additionally, outcome measures like the presence of dactylitis and enthesitis were included.

### Conventional radiography

Radiographs of both hands and feet were evaluated using the PsA-modified SHS score to quantify erosion and joint space narrowing (JSN) [5]. Erosion was scored on a scale of 0–3 (none/discrete erosion/large erosion not passing midline/large erosion passing midline) and JSN on a scale of 0–4 (normal/asymmetrical or minimal narrowing up to 25%/definite narrowing with loss of up to 50% of the normal space/definite narrowing with loss of 50–99% of the normal space or subluxation/absence of a joint space, presumptive evidence of ankyloses, or complete subluxation). Scoring was done by one observer (musculoskeletal radiologist) blinded for clinical information. Scores of hands and feet were summed to achieve total erosion and JSN scores. Thus, the maximum scores were 208 (160 hands, 48 feet) and 80 (44 hands, 36 feet) for JSN and erosion, respectively.

### MRI: HEMRIS and PsAMRIS scores

MRI scans of both ankles were performed with a field strength 1.5 or 3 T MR equipment and an extremity coil. The MRI protocol adhered to the European Society of Musculoskeletal Radiology recommendations and included the following sequences: 3D proton density with fat suppression (FS), transversal T1 turbo spin echo and 3D T1 FS before and after intravenous gadolinium injection [16]. Ankle MRIs were visually assessed with the HEMRIS evaluation using inflammatory and structural pathologies at the site of the entheses of the Achilles tendon and plantar fascia [7, 8]:

- HEMRIS inflammation (scale: 0–21):
  - Achilles tendon (scale: 0–3 for each pathology): Achilles tendon intratendon hypersignal, Achilles tendon peritendon hypersignal, Achilles tendon bone marrow oedema and Achilles tendon retrocalcaneal bursitis.
  - Plantar fascia (scale: 0–3 for each pathology): Plantar fascia bone marrow oedema, plantar fascia periaponeurosis hypersignal and plantar fascia intraaponeurosis hypersignal.
- HEMRIS structure (scale: 0–18):
  - Achilles tendon (scale: 0–3 for each pathology): Achilles tendon thickness, Achilles tendon bone spur and Achilles tendon bone erosion.
  - Plantar fascia (scale: 0–3 for each pathology): Plantar fascia bone spur, plantar fascia bone erosion and plantar fascia tendon thickness.

Total inflammation and structure scores were achieved by adding plantar fascia and Achilles tendon scores and used for analysis. Also, the separate scores for the Achilles tendon and plantar fascia were analysed for a detailed assessment.

PsAMRIS [7, 8], adapted for the heel, was used to evaluate synovial enhancement, tenosynovitis, periarticular bone oedema and erosions:

- PsAMRIS synovial enhancement (scale: 0–3 for each pathology): Synovial enhancement of anterior ankle, posterior ankle, tarsal sinus and midfoot.
- PsAMRIS tenosynovitis (scale: 0–3 for each pathology): Tenosynovitis of tibialis posterior, flexor digitorium longus, flexor halluxis longus tibialis and peroneal tendons.
- PsAMRIS bone erosion (scale: 0–10 for each pathology): Periarticular bone erosion of tibia, fibula, talus and calcaneus.
- PsAMRIS bone oedema (scale: 0–10 for each pathology): Periarticular bone oedema of tibia, fibula, talus and calcaneus.

All the measures were scored by two independent musculoskeletal radiologists blinded for clinical information. Subsequent consensus readings were performed in cases of disagreement. The indicated pathologies were summated per ankle to be averaged between the left and right ankle. Thus, final scores reflect both ankles, with a maximum of 21 for HEMRIS inflammation (12 for Achilles tendon and 9 for plantar fascia), 18 for HEMRIS structure (9 for Achilles tendon and 9 for plantar fascia), 12 for PsAMRIS synovial enhancement and tenosynovitis, 40 for PsAMRIS bone erosion and oedema.

### Pet/ct

Whole body ^18^F-FDG PET/CT was performed after overnight fasting and one hour after intravenous administration of fluorodeoxyglucose (^18^F-FDG). For co-registration and attenuation correction, a non-contrast-enhanced low-dose CT was obtained. To ensure repeatability and reproducibility of quantitative PET/CT outcome measures, PET/CT reconstructions were executed following the guideline of the European Association of Nuclear Medicine Research Ltd (EARL) [17]. Afterward, the quality of PET/CT scans were assessed, and synovitis was scored (based on the Deauville scale [18]) from PET/CT scans of shoulder, elbow, carpus, hip, knee and ankle. All the pathologies were scored on a scale of 0–4 (no enhanced uptake/slight uptake, but below blood pool/uptake above mediastinal, but < liver/uptake moderately > liver/uptake > three times liver uptake). Scoring was completed by one observer (nuclear radiologist) blinded for clinical information. At the end, all joint scores were summated to obtain one synovitis score with a maximum of 48.

Vascular inflammation of the aortic wall was calculated to evaluate systemic inflammation. Target-to-background ratios (TBR) were used to assess aortic vascular inflammation in a reliable and reproducible manner [11, 12, 19]. Two-dimensional region of interests (ROIs) were manually drawn on PET/CT scans around the external aortic contour in the axial setting using IntelliSpace software. ROIs were placed along the aorta on every slice that was visible to acquire maximum standardized uptake value (SUV). The SUVmax values per slice along the aorta were averaged to obtain SUVmax for the entire aorta and per aortic segment (ascending aorta, aortic arch, descending aorta, suprarenal abdominal aorta and infrarenal abdominal aorta [12]). Background activity SUVmean was derived from averaging at least six ROIs in the superior vena cava (SVC), or, in one case, at the inferior vena cava due to visual spill of activity at the myocardium. Subsequently, the maximum TBR of the aorta was calculated by dividing SUVmax of the aorta by SUVmean of the SVC [17, 20]. The same approach was used for the calculation of maximum TBR per aortic segments.

### Classification of imaging parameters

All the parameters that were derived from medical images were classified as inflammatory or structural imaging parameters as shown below:

- Inflammatory imaging parameters: aortic vascular inflammation (TBR), PET/CT synovitis, HEMRIS inflammation, HEMRIS inflammation Achilles tendon, HEMRIS inflammation plantar fascia, PsAMRIS synovial enhancement, PsAMRIS tenosynovitis and PsAMRIS bone oedema.
- Structural imaging parameters: HEMRIS structure, HEMRIS structure Achilles tendon, HEMRIS structure plantar fascia, PsAMRIS bone erosion, SHS erosion and SHS joint space narrowing.

### Statistical analysis

Descriptive statistics [median with interquartile range (IQR) for continuous and non-normally distributed variables, mean S.D. for continuous and normally distributed variables, frequencies with percentages for categorical variables] were used to summarize baseline patient characteristics. In cases where scans were performed but part of the sub-scores were missing, single imputation was performed using the SPSS imputation algorithm (linear regression) with the constraint that the imputed values could not be lower than 0. Since most of the imaging total scores were non-normally distributed according to Q-Q plots, all imaging scores were logarithmically (natural log) transformed before statistical evaluation to normalize the distribution and have an interpretable meaning.

Patient characteristics were compared between groups using the independent *t* test for normally distributed variables, Mann–Whitney U for non-normally distributed variables, and a χ^2^ test for categorical variables. Each imaging parameter was compared between groups with a univariable analysis using linear regression where the (log transformed) imaging parameters as dependent variable and grouping as independent variable. To ensure averaging between right and left of the HEMRIS and PsAMRIS scores did not affect results, sensitivity analyses were performed and the maximum scores of both sides were plotted. For imaging parameters shown to be different between groups (*P* < 0.1), sub-scores were evaluated separately. Based on the *P*-value (*P* < 0.1), imaging parameters were evaluated with a multivariable analysis using multiple linear regression to inspect the influence of patient characteristics on this difference. Grouping and clinical and demographic variables were included as independent variables and the imaging parameter (log transformed) as the dependent variable.

Among all patient characteristics, six clinical and demographic variables were chosen for the multivariable analysis mentioned above: gender, age, BMI, smoking status, time since diagnosis of PsA and time since diagnosis of psoriasis. These were chosen based on *P*-value, literature and clinical experts in combination [21–24]. The clinical and demographic variables that had significant *P*-values were also identified as important by our team of clinical experts, ensuring their clinical relevance and significance. Among these variables, smoking status was combined as ever smokers (current and ex-smokers grouped) and never-smokers (patients who have never smoked) for the analyses. Furthermore, clinical and demographic variables were considered confounding and left in the optimized multiple linear regression model if they changed the effect estimate (unstandardized B) of the grouping variable by 10% or more. Statistical analyses were performed using SPSS version 27 (IBM SPSS Statistics, IBM Corporation, Armonk, NY, USA) and the significance level was set at *P* < 0.05.

## Results

### Patient characteristics

All participants had at least one type of available imaging data and parameters. For DMARD-naive patients, three MRI and one PET/CT datasets were missing whereas this was five MRI and three PET/CT datasets for DMARD-failure patients. All conventional radiograph data were present for every patient. Detailed information related to the number of missing data for each sub-score is given in Supplementary Table S3, available at *Rheumatology* online. The majority of the patient characteristics were comparable between the groups (Table 1). However, DMARD-naive patients were on average younger and had a shorter disease duration (time since diagnosis of PsA and psoriasis *P* < 0.008).

**Table 1.** Baseline patient characteristics given in mean (S.D.), median (IQR) or frequencies (%) and their *P*-values

	DMARD-naive	DMARD-failure	
Patient characteristics	(*n* = 40)	(*n* = 40)	*P*-values^e^
***Clinical and demographic variables***			
Age, years [median (IQR)]	48.9 (45.1–58)	55.4 (49.5–60.8)	**0.013**
Female, *n* (%)	18 (45)	15 (37.5)	0.496
BMI (kg/m^2), mean (S.D.)	28.3 (5.4)	28.2 (4.3)	0.963
Smoking status, *n* (%)	—	—	0.181
Smoker	7 (17.5)	2 (5)	—
Ex-smoker	16 (40)	16 (40)	—
Never smoked	17 (42.5)	22 (55)	—
Time since diagnosis of psoriatic arthritis (years), median (IQR)	0.1 (0.1–0.8)	7.4 (2.4–17.1)	**<0.001**
Time since diagnosis of psoriasis (years), median (IQR)	2.9 (0.3–18.6)	11.5 (5.6–25)	**0.007**
***Disease-related variables***			
PASI (Psoriasis Area Severity Index), median (IQR)	1.5 (0.6–4.8)	1.1 (0–2.4)	0.168
CRP (mg/L), median (IQR)	3.5 (1–11.5)	3.2 (1–9.5)	0.835
HAQ [median (IQR)]	0.6 (0.3–1.3)	0.7 (0.4–0.9)	0.820
Presence of dactylitis currently, *n* (%)^a^	10 (25)	10 (25)	1
Presence of enthesitis currently (LEI), *n* (%)^a^	9 (22.5)	12 (30)	0.446
***Medication use***			
History use of DMARD, *n* (%)	—	—	—
None	40 (100)	0 (0)	**<0.001**
csDMARD^b^	0 (0)	40 (100)	**<0.001**
bDMARD^c^	0 (0)	4 (10)	**0.040**
History use of prednisone, *n* (%)	7 (17.5)^d^	12 (30)	0.189
Current use of medication	—	—	—
Methotrexate, *n* (%)	0 (0)	32 (80)	**<0.001**
Methotrexate median (IQR) dosage (mg/week)	0 (0–0)	20 (15–25)	—
Leflunomide, *n* (%)	0 (0)	5 (12.5)	**0.021**
Leflunomide median (IQR) dosage (mg/day)	0 (0– 0)	20 (20–20)	—
Sulfasalazine, *n* (%)	0 (0)	3 (7.5)	0.077
Sulfasalazine median (IQR) dosage (mg/day)	0 (0–0)	3000 (2000–3000)	—
Daily use of NSAID, *n* (%)	21 (52.5)	18 (45)	0.502

a Presence of dactylitis and enthesitis were determined by the physician.

b csDMARD group consists of: sulfasalazine, leflunomide, hydroxychloroquine and methotrexate.

c bDMARD groups consists of: golimumab, adalimumab, infliximab, certolizumab, secukinumab, ixekizumab and ustekinumab.

d In terms of the oral use of corticosteroid for DMARD-naive patients, a stable dose of ≤10 mg/day of prednisone (or equivalent) for ≥4 weeks prior to baseline visit was allowed.

e Bold p-values values indicate *P* < 0.05, which is considered statistically significant.

BMI: body mass index; CRP: C-reactive protein; HAQ: health assessment questionnaire; LEI: Leeds enthesitis index; DMARD: disease-modifying antirheumatic drug; csDMARD: conventional synthetic disease-modifying antirheumatic drug; bDMARD: biological disease-modifying antirheumatic drug; NSAID: non-steroid anti-inflammatory drug.

### Univariable analysis: differences in imaging parameters

**Figure 1.**
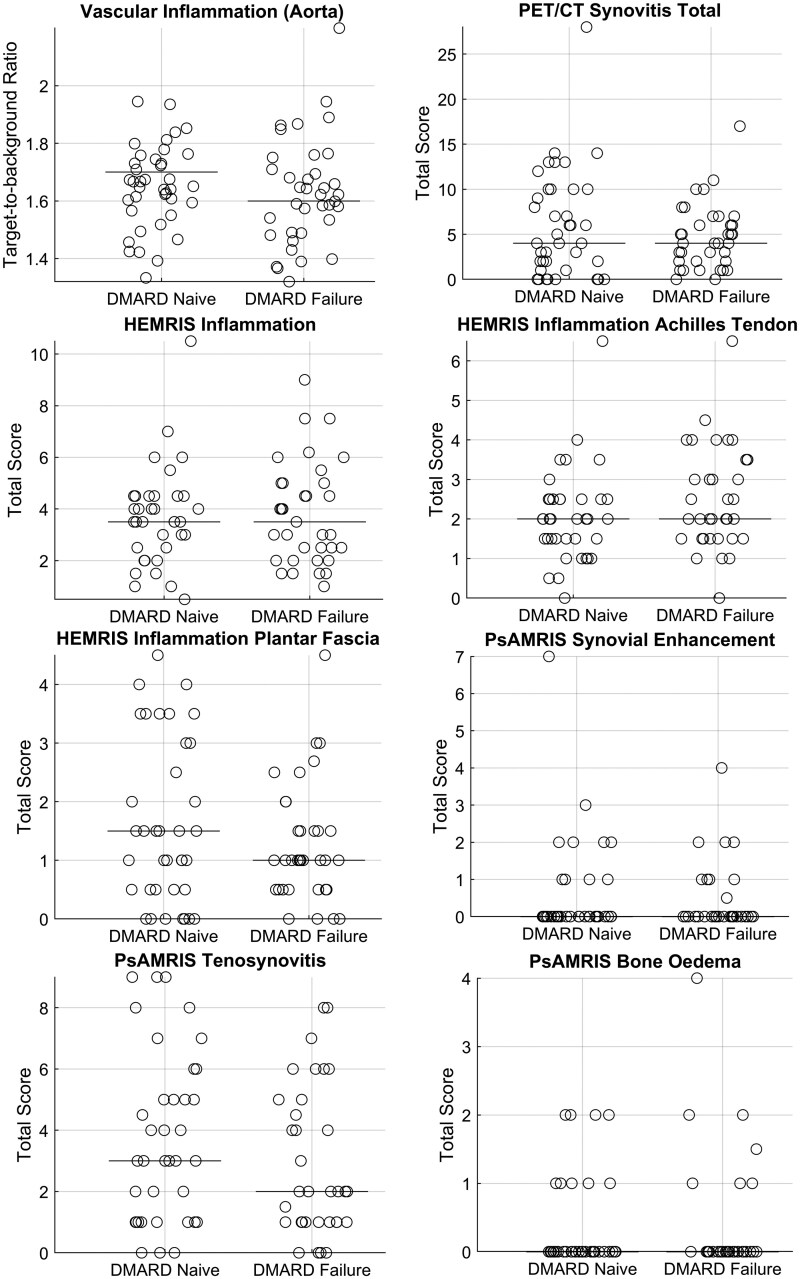
Illustration of between-group differences for inflammatory imaging parameters. Each circle represents an individual, and the line represents the median value of the group

**Figure 2.**
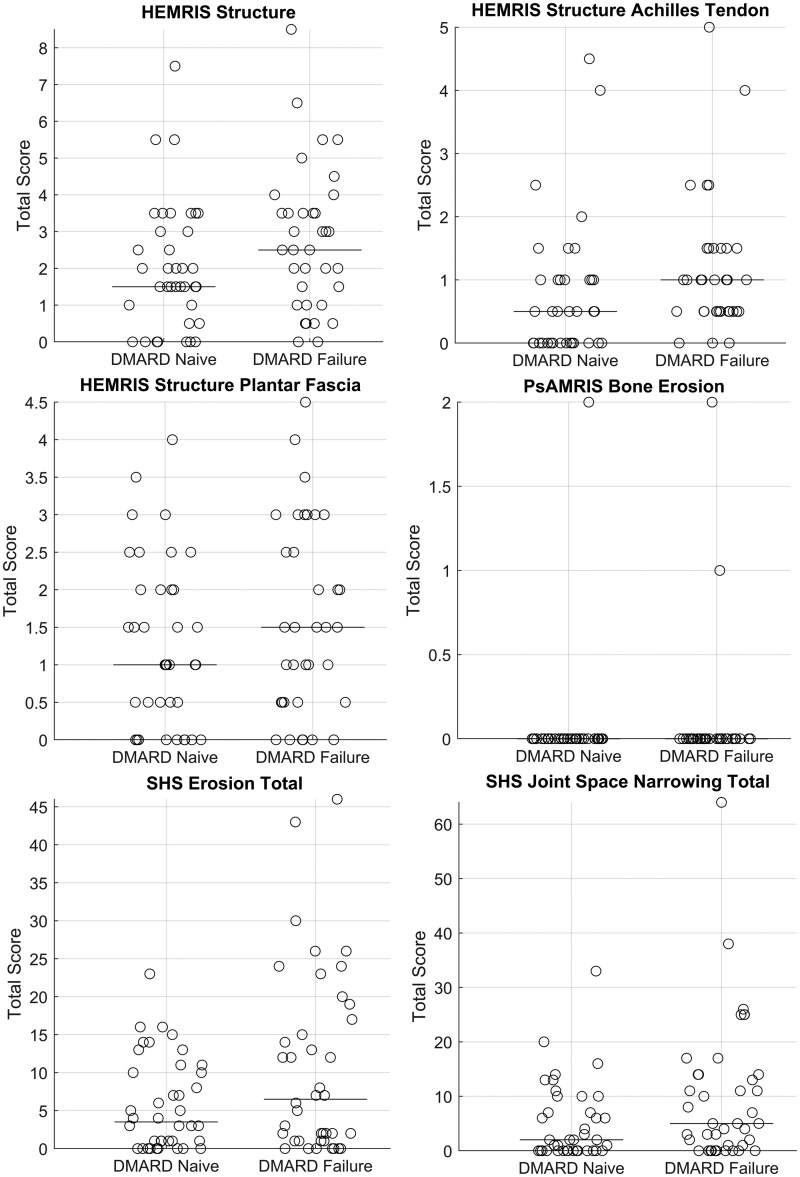
Illustration of between-group differences for structural imaging parameters. Each circle represents an individual, and the line represents the median value of the group. HEMRIS structure Achilles tendon (*P* = 0.024) was statistically significant whereas SHS JSN (*P* = 0.050) was not significant

Generally, observed values for inflammation (Fig. 1) and structural damage (Fig. 2) were low, considering the maximum of each imaging score. Most of the results did not differ significantly between the patient groups (Table 2). Inflammatory parameters seemed to be slightly higher in DMARD-naive patients, whereas for structural damage, DMARD-failure patients showed somewhat higher values (Figs 1 and 2). The majority of the patients had some sort of inflammation or structural damage (Supplementary Table S1, available at *Rheumatology* online). Among all the imaging parameters, only HEMRIS structure Achilles tendon was significantly different between groups (*P* = 0.024, R^2^ = 0.071), while SHS JSN was not statistically significant (*P* = 0.050, R^2^ = 0.048), with higher values in DMARD-failure for both parameters. These observed differences were not clearly explained by one specific sub-score (Supplementary Figs S1 and S2, available at *Rheumatology* online). For HEMRIS and PsAMRIS, using maximum scores instead of average scores between left and right did not change results (Supplementary Figs S3 and S4, available at *Rheumatology* online). Therefore, the averaged scores were used in all analyses.

**Table 2.** The difference between the groups in terms of imaging parameters and their *P*-values

	DMARD-naive	DMARD-failure	
	(*n* = 40)	(*n* = 40)	
Imaging parameters	Median(IQR)^a^	Median(IQR)^a^	*P*-Values^b^^c^
Inflammatory imaging parameters	—	—	—
TBR aorta	1.7 (1.6–1.7)	1.6 (1.5–1.7)	0.534
PET/CT synovitis total (0–60)	4 (1–10)	4 (2–6.5)	0.984
HEMRIS inflammation (0–21)	3.5 (2.5–4.5)	3.5 (2–5)	0.854
HEMRIS inflammation achilles tendon (0–12)	2 (1.5–2.5)	2 (1.5–3.5)	0.125
HEMRIS inflammation plantar fascia (0–9)	1.5 (0.5–3)	1 (0.5–1.5)	0.438
PsAMRIS synovial enhancement (0–12)	0 (0–1)	0 (0–0.9)	0.770
PsAMRIS tenosynovitis (0–12)	3 (1–5.5)	2 (1–5)	0.223
PsAMRIS bone oedema (0–40)	0 (0–0.5)	0 (0–0)	0.899
Structural imaging parameters	—	—	—
HEMRIS structure (0–18)	1.5 (0.8–3.3)	2.5 (1–3.5)	0.090
HEMRIS structure achilles tendon (0–9)	0.5 (0–1)	1 (0.5–1.5)	**0.024**
HEMRIS structure plantar fascia (0–9)	1 (0.3–2)	1.5 (0.5–3)	0.311
PsAMRIS bone erosion (0–40)	0 (0–0)	0 (0–0)	0.630
SHS erosion (0–80)	3.5 (0.3–10.8)	6.5 (1–18.5)	0.128
SHS joint space narrowing (0–208)	2 (0–9.3)	5 (0.3–14)	0.050

a Median (IQR) data were presented for non-natural logarithm transformed data.

b The *P*-values were calculated from natural logarithm transformed data using linear regression to find the difference between the groups. The same linear regression approach as the following calculations were used to help interpretation of the results.

c Bold p-values values indicate *P* < 0.05, which is considered statistically significant.

HEMRIS: Heel Enthesitis Magnetic Resonance Imaging Scoring System; PsAMRIS: Psoriatic Arthritis Magnetic Resonance Imaging Scoring System [adapted for the ankle]; SHS: Sharp–van der Heijde; TBR: target-to-background ratio (TBR).

### Multivariable analysis: influence of patient characteristics

Multiple linear regression showed that several clinical and demographic variables were confounders and the differences between groups in HEMRIS structure Achilles tendon and SHS JSN were influenced by these confounders (Table 3). For HEMRIS structure Achilles tendon and SHS JSN, confounders in the optimized models are given in Table 3. After correcting for the confounders, the imaging parameters were no longer significantly different between groups (both *P* > 0.600). Older patients had significantly more JSN (*P* < 0.001), and ever-smoking patients had significantly more structural damage at Achilles tendon (*P* = 0.037). Remaining confounders did not influence the imaging parameters significantly (Table 3).

**Table 3.** Multiple linear regression analysis results: confounder clinical and demographic variables of the optimized model are given under the designated imaging parameter

	Before clinical and demographic variable correction	After clinical and demographic variable correction	
	*P*-value	*P*-value^a^	Standardized coefficients beta
HEMRIS structure Achilles tendon	—	—	—
Grouping (DMARD-naive or DMARD-failure)	**0.024**	0.711	0.050
Time since diagnosis of PsA	—	0.177	0.183
Ever smoking	—	**0.037**	0.247
SHS joint space narrowing	—	—	—
Grouping (DMARD-naive or DMARD-failure)	0.050	0.982	0.002
Time since diagnosis of PsA	—	0.071	0.223
Ever smoking	—	0.560	−0.057
Age	—	**<0.001**	0.414
BMI	—	0.134	0.147
Gender	—	0.174	−0.132

DMARD: disease-modifying antirheumatic drug; HEMRIS: Heel Enthesitis Magnetic Resonance Imaging Scoring System; PsA: psoriatic arthritis; SHS: Sharp–van der Heijde.

a Bold p-values values indicate *P* < 0.05, which is considered statistically significant.

## Discussion

To our knowledge, this study is the first study that combines various imaging approaches to analyse the heterogeneous manifestations of PsA by comparing two patient groups with active peripheral arthritis, namely DMARD-naive and DMARD-failure. These groups were compared by evaluating whether they differ in inflammatory and structural imaging parameters. The results showed that structural damage and inflammation scores were similar between the groups, especially after adjusting for clinical and demographic variables. This result implies that the DMARD-failure patient group was not associated with worsened inflammatory and structural imaging findings in our study.

Only HEMRIS structure Achilles tendon was significantly different between groups, while SHS JSN was not statistically significant, with higher values in DMARD-failure for both parameters, which indicates more structural damage. As there are no established minimum clinically important differences (MCIDs) for these imaging scores, we provided effect sizes (R^2^) for HEMRIS and SHS JSN scores and relied on clinician expert opinion to interpret clinical relevance [25]. Based on this, the clinical relevance of these differences is doubtful, as between-group differences were very low (∼0.5 on a scale 0–9 for HEMRIS structure Achilles tendon and 3.0 on a scale 0–208 for SHS JSN). Furthermore, after correcting for confounding clinical and demographic variables, the differences between the groups disappeared. To ensure that the lack of differences between groups was not the result of our averaging approach between joints, sensitivity analyses for HEMRIS and PsAMRIS were performed to validate our findings. The results were similar with averaging approach and confirmed that our original findings were robust. Moreover, the regression analysis revealed that older patients exhibit significantly more JSN and patients who ever smoked had more structural damage at the Achilles tendon. However, the case related to JSN may be due to other factors such as overuse of joints or ageing rather than reflecting the severity of PsA.

We considered whether our lack of differences between DMARD-failure and naive patients was due to our cohort’s lower disease activity compared with literature in terms of inflammation and structural damage, which is crucial for interpreting our results and statistical findings. Our systematic literature search revealed that patient characteristics, and inflammation markers such as CRP were in a similar range (Supplementary Table S2 [28–35], available at *Rheumatology* online) compared with existing PsA cohorts. Finally, although there were only a few publications, values for TBR [26] and HEMRIS [27] were found to be very well-matched. Therefore, our cohort seems to be a representative cohort for PsA and our re-assuring findings likely generalize to PsA patients.

A major strength of this study is the involvement of multimodal imaging and scores, capturing diverse inflammatory and structural aspects of PsA, and broadening our understanding of its heterogeneous nature. Most PsA studies use one imaging method such as MRI or conventional radiograph, whereas ours uses several (conventional radiograph, MRI and PET/CT) to capture different characteristics. Moreover, the comparison of PsA patient groups with prior DMARD use has not been investigated. Our study addresses this gap by categorizing patients into two groups: PsA patients who never used a DMARD compared with patients who failed on DMARD. These findings can be useful in clinical practice by providing insights into disease progression and disease characteristics for treatment decisions. Comparing the groups revealed that failing a DMARD may not lead to increased inflammation or structural damage.

Despite its strength, our study has certain limitations. Firstly, the sample size of 80 patients might not capture the full diversity of PsA, although validation using the TOFA-PREDICT validation cohort (another group of 80 patients) is possible. Because the commonly affected joints are the hands and feet, followed by knees, wrists, ankles and shoulders [4], we analysed these joints using various imaging techniques. However, other locations like the spine and sacroiliac joints were not included, which could be a limitation. While we evaluated numerous joints with radiographs and PET/CT, only the ankles were evaluated with MRI, which may have limited our ability to capture the full spectrum of disease manifestation. To overcome this limitation to some extent, TBR was used as a more general measure of inflammation. In addition, PsAMRIS, adapted for ankles, is an approach that was not validated, which constitutes a potential limitation, as the reliability and accuracy of this modified method have not been established yet. The absence of ultrasonography and corresponding imaging scores is also a limitation, as it could enhance the assessment of structural damage and inflammation.

Furthermore, our inclusion criteria focused on PsA patients with active peripheral arthritis (≥2 swollen joints and ≥2 tender joints), limiting the generalizability of our findings to this domain rather than the full PsA spectrum. Also, we assessed disease activity in our patient cohort using imaging scores to ensure our results’ generalizability, but we did not focus on how disease activity duration and extent affect structural damage or inflammation. Prolonged inflammation may lead to greater structural damage over time, potentially resulting in underestimated structural damage and inflammation. Similarly, our analyses did not include comorbidities like diabetes and cardiovascular disease, or type and duration of previous medication use, which may impact structural damage and inflammation.

In conclusion, DMARD-naive and DMARD-failure PsA patients (unresponsive to csDMARD, but one prior bDMARD excluding etanercept was allowed) with active peripheral arthritis showed similar inflammation and structural damage on imaging, especially after the correction of clinical and demographic variables. Thus, DMARD-naive and DMARD-failure patient groups may be combined in future PsA progression and treatment decision studies.

## Supplementary Material

keae450_Supplementary_Data

## Data Availability

The data underlying this article will be shared on reasonable request to the corresponding author.
