# Supplementary material for: Multimodal imaging of structural damage and inflammation in psoriatic arthritis: a comparison of DMARD-naive and DMARD-failure patients
Source: Rheumatology (Oxford). 2024 Aug 17;64(4):1760–9. doi: 10.1093/rheumatology/keae450 (PMC11962931; doi:10.1093/rheumatology/keae450)
Supplement: keae450_Supplementary_Data [file keae450_supplementary_data.zip › keae450_Supplementary_Data/rhe-24-0820-File005.docx]

Supplementary Table S1

|  | **DMARD-Naive**  **(n=40)** | **DMARD-Failure**  **(n=40)** |
| --- | --- | --- |
| Median (IQR) | - | - |
| TBR Vascular Inflammation | - | - |
| Ascending Aorta | 1.7 (1.6 - 1.8) | 1.7 (1.5 - 1.8) |
| Aortic Arch | 1.7 (1.5 - 1.8) | 1.6 (1.5 - 1.8) |
| Descending Aorta | 1.7 (1.6 - 1.8) | 1.6 (1.5 - 1.8) |
| Suprarenal Abdominal Aorta | 1.7 (1.6 - 1.8) | 1.6 (1.4 - 1.7) |
| Infrarenal Abdominal Aorta | 1.6 (1.4 - 1.6) | 1.6 (1.5 - 1.7) |
| PET/CT Synovitis | - | - |
| Shoulder | 2 (0 - 3) | 2 (1 - 2) |
| Elbow | 0 (0 - 0) | 0 (0 - 0) |
| Carpus | 0 (0 - 2) | 0 (0 - 1) |
| Hip | 0 (0 - 0) | 0 (0 - 0) |
| Knee | 2 (0 - 4) | 2 (0 - 3) |
| Ankle | 0 (0 - 0) | 0 (0 - 0) |
| SHS | - | - |
| Hand Erosion | 3.5 (0 - 8) | 3.5 (0 - 12) |
| Feet Erosion | 0 (0 - 1) | 1.5 (0 - 5.8) |
| Hand JSN | 1 (0 - 7) | 3 (0 - 9.8) |
| Feet JSN | 0 (0 - 2) | 2 (0 - 5) |
| HEMRIS Structure | - | - |
| Achilles Tendon Thickness | 0 (0 - 0.5) | 0 (0 - 0.5) |
| Achilles Tendon Bone Spur | 0 (0 - 0.5) | 0.5 (0 - 1) |
| Achilles Tendon Bone Erosion | 0 (0 - 0) | 0 (0 - 0) |
| Plantar Fascia Tendon Thickness | 0.5 (0.5 - 1.3) | 0.5 (0 - 1) |
| Plantar Fascia Bone Spur | 0.5 (0 - 1) | 1 (0 - 1.5) |
| Plantar Fascia Bone Erosion | 0 (0 - 0) | 0 (0 - 0) |
| HEMRIS Inflammation | - | - |
| Achilles Tendon Retrocalcaneal Bursitis | 0.5 (0 - 0.8) | 0.5 (0 - 1) |
| Achilles Tendon Peritendon Hypersignal | 0.5 (0 - 1) | 0.5 (0 - 1) |
| Achilles Tendon Intratendon Hypersignal | 1 (0.8 - 1) | 1 (1 - 1.5) |
| Achilles Tendon Bone Marrow Oedema | 0 (0 - 0) | 0 (0 - 0) |
| Plantar Fascia Bone Marrow Oedema | 0 (0 - 0.8) | 0.5 (0 - 1) |
| Plantar Fascia Periaponeurosis Hypersignal | 0.5 (0 - 1) | 0.5 (0 - 0.5) |
| Plantar Fascia Intraaponeurosis Hypersignal | 0.5 (0 - 1) | 0 (0 - 1) |
| PsAMRIS Synovial Enhancement | - | - |
| Anterior Ankle | 0 (0 - 0) | 0 (0 - 0) |
| Posterior Ankle | 0 (0 - 0) | 0 (0 - 0) |
| Tarsalsinus | 0 (0 - 0) | 0 (0 - 0) |
| Midfoot | 0 (0 - 0) | 0 (0 - 0) |
| PsAMRIS Tenosynovitis | - | - |
| Tibialis Posterior | 1 (1 - 2) | 1 (1 - 2) |
| Peroneal Tendons | 0 (0 - 1) | 0 (0 - 1) |
| Flexor Digitorium Longus | 0 (0 - 1) | 0 (0 - 1) |
| Flexor Halluxis Longus Tibialis | 1 (0 - 3) | 0 (0 - 2) |
| PsAMRIS Bone Erosion | - | - |
| Tibia | 0 (0 - 0) | 0 (0 - 0) |
| Fibula | 0 (0 - 0) | 0 (0 - 0) |
| Talus | 0 (0 - 0) | 0 (0 - 0) |
| Calcaneus | 0 (0 - 0) | 0 (0 - 0) |
| PsAMRIS Bone Oedema | - | - |
| Tibia | 0 (0 - 0) | 0 (0 - 0) |
| Fibula | 0 (0 - 0) | 0 (0 - 0) |
| Talus | 0 (0 - 0) | 0 (0 - 0) |
| Calcaneus | 0 (0 - 0) | 0 (0 - 0) |

Supplementary Table S2

|  | | | | | | | | | | | | | |
| --- | --- | --- | --- | --- | --- | --- | --- | --- | --- | --- | --- | --- | --- |
|  | **Present study** | | | **Araujo** [28] | **Wells** [29] | **Kane** [30] | **Mease** [31] | **Gladman** [32] | | **Shin** [33] | | **Zisman** [34] | **Szentrpetery** [35] |
| **Patient profile** | Total 80 active PsA patients | 40 DMARD-naive patients | 40 DMARD-failure patients | PsA patients who used DMARDs | csDMARD naive and biologic naive PsA patients | Early PsA patients | PsA patients at baseline receiving biologics and/or targeted synthetic DMARDs | Early PsA patients with 2 groups. Group 1 (within 2 years of diagnosis) and group 2 (disease duration greater than 2 years). | | | PsA patients in Korea | PsA patients in Israel | Recent-onset (<12 months), treatment naive PsA |
| **Number of patients** | 80 | 40 | 40 | 26 | 527 | 129 | 148 | 436 | 641 | 22 | | 149 | 32 |
| **Age (years) (mean)** | 51.3 | 48.5 | 54.1 | 55.2 | 49.4 | 41.2 | 54.7 | - | - | 42.2 | | 58.2 | 40 |
| **Female (%)** | 41.3 | 45 | 37.5 | 23.1 | 52.6 | 47.3 | 54 | 42.4 | 44.8 | 54.5 | | 57 | 46.9 |
| **BMI (kg/m^2) (mean)** | 28.3 | 28.3 | 28.2 | 26.5 | 29.1 | - | 33.1 | - | - | ≥ 25 (for 11 patients) | | - | - |
| **Time since diagnosis of Arthritis Psoriasis (years) (mean)** | 6.2 | 1.7 | 10.8 | 6.5 | 3.4 | <1 years (9.9 months) | 11.8 | 0.9 | 11 | 5.9 | | - | - |
| **Time since diagnosis of Psoriasis (years) (mean)** | 13.8 | 11.1 | 16.5 | - | 15.8 | - | - | - | - | 10.8 | | 15.5 | - |
| **PASI (mean)** | 3.2 | 3.5 | 2.9 | 0.2 | 7.2 (Scale[0-72]) | - | - | 6.2 | 5.5 | - | | - | 3.3 (0 - 27.7) median |
| **CRP (mg/L) (mean)** | 8.6 | 10.4 | 6.9 | 1.8 | 9 | 27.6 (for 112 patients) | 4.3 | - | - | - | | above 5 (for 86 out of 123 patients) | 6.6 |
| **HAQ (mean)** | 0.8 | 0.8 | 0.7 | 0.2 | 1.1 | 0.7 (completed by 74 patients) | - | - | - | - | | - | 0.6 |
| **Presence of dactylitis (percentages)** | 25 | 25 | 25 | - | 65.1 | 28.7 | 13.5 | - | - | 22.7 | | - | 31.2 |
| **Presence of enthesitis (percentages)** | 26.3 | 22.5 | 30 | - | 490.9 | - | 31.7 | - | - | - | | - | - |
| **RF negative (number)** | 75 | 37 | 38 | - | - | 124 | - | - | - | - | | - | 32 |
| **ESR (mean)** | 15.5 | 19.4 | 11.6 | 8.6 | - | 24 (for 124 patients) | 17.1 | - | - | - | | - | 12 |

Supplementary Table S3

| **Imaging Sub-Scores** | **Not Missing Cases** | **Missing** |  |
| --- | --- | --- | --- |
|  | **Count** | **Count** | **Percent** |
| psamris synovialenhancement anteriorankle left^a^ | 69 | 11 | 13.7 |
| psamris synovialenhancement posteriorankle left^a^ | 69 | 11 | 13.7 |
| psamris synovialenhancement tarsalsinus left ^a^ | 69 | 11 | 13.7 |
| psamris synovialenhancement midfoot left^a^ | 51 | 29 | 36.3 |
| psamris tenosynovitis tibialis posterior left^a^ | 71 | 9 | 11.3 |
| psamris flexor digitorium longus left^a^ | 71 | 9 | 11.3 |
| psamris flexor halluxis longus tibialis left^a^ | 71 | 9 | 11.3 |
| psamris peroneal tendons left^a^ | 71 | 9 | 11.3 |
| psamris boneerosion tibia left^a^ | 71 | 9 | 11.3 |
| psamris boneerosion fibula left^a^ | 71 | 9 | 11.3 |
| psamris boneerosion talus left^a^ | 69 | 11 | 13.7 |
| psamris boneerosion calcaneus left^a^ | 68 | 12 | 15 |
| psamris boneedema tibia left^a^ | 71 | 9 | 11.3 |
| psamris boneedema fibula left^a^ | 71 | 9 | 11.3 |
| psamris boneedema talus left^a^ | 68 | 12 | 15 |
| psamris boneedema calcaneus left^a^ | 66 | 14 | 17.5 |
| psamris synovialenhancement anteriorankle right^a^ | 65 | 15 | 18.8 |
| psamris synovialenhancement posteriorankle right^a^ | 65 | 15 | 18.8 |
| psamris synovialenhancement tarsalsinus right^a^ | 65 | 15 | 18.8 |
| psamris synovialenhancement midfoot right^a^ | 48 | 32 | 40 |
| psamris tenosynovitis tibialis posterior right^a^ | 67 | 13 | 16.2 |
| psamris flexor digitorium longus right^a^ | 67 | 13 | 16.2 |
| psamris flexor halluxis longus tibialis right^a^ | 67 | 13 | 16.2 |
| psamris peroneal tendons right^a^ | 67 | 13 | 16.2 |
| psamris boneerosion tibia right^a^ | 67 | 13 | 16.2 |
| psamris boneerosion fibula right^a^ | 67 | 13 | 16.2 |
| psamris boneerosion talus right^a^ | 65 | 15 | 18.8 |
| psamris boneerosion calcaneus right^a^ | 64 | 16 | 20 |
| psamris boneedema tibia right^a^ | 67 | 13 | 16.2 |
| psamris boneedema fibula right^a^ | 67 | 13 | 16.2 |
| psamris boneedema talus right^a^ | 64 | 16 | 20 |
| psamris boneedema calcaneus right^a^ | 62 | 18 | 22.5 |
| hemris achilles tendon thickness left^a^ | 70 | 10 | 12.5 |
| hemris achilles tendon retrocalcaneal bursitis left^a^ | 68 | 12 | 15 |
| hemris achilles tendon peritendon hypersignal left^a^ | 69 | 11 | 13.7 |
| hemris achilles tendon intratendon hypersignal left^a^ | 69 | 11 | 13.7 |
| hemris achilles tendon bone spur left^a^ | 70 | 10 | 12.5 |
| hemris achilles tendon bone erosion left^a^ | 69 | 11 | 13.7 |
| hemris achilles tendon bone marrow oedema left^a^ | 69 | 11 | 13.7 |
| hemris plantar fascia tendon thickness left^a^ | 71 | 9 | 11.3 |
| hemris plantar fascia periaponeurosis hypersignal left^a^ | 63 | 17 | 21.3 |
| hemris plantar fascia bone spur left^a^ | 70 | 10 | 12.5 |
| hemris plantar fascia bone erosion left^a^ | 71 | 9 | 11.3 |
| hemris plantar fascia bone marrow oedema left^a^ | 58 | 22 | 27.5 |
| hemris plantar fascia Intraaponeurosis hypersignal left^a^ | 67 | 13 | 16.2 |
| hemris achilles tendon thickness right^a^ | 70 | 10 | 12.5 |
| hemris achilles tendon retrocalcaneal bursitis right^a^ | 69 | 11 | 13.7 |
| hemris achilles tendon peritendon hypersignal right^a^ | 69 | 11 | 13.7 |
| hemris achilles tendon intratendon hypersignal right^a^ | 69 | 11 | 13.7 |
| hemris achilles tendon bone spur right^a^ | 70 | 10 | 12.5 |
| hemris achilles tendon bone erosion right^a^ | 69 | 11 | 13.7 |
| hemris achilles tendon bone marrow oedema right^a^ | 68 | 12 | 15 |
| hemris plantar fascia tendon thickness right^a^ | 69 | 11 | 13.7 |
| hemris plantar fascia periaponeurosis hypersignal right^a^ | 64 | 16 | 20 |
| hemris plantar fascia bone spur right^a^ | 69 | 11 | 13.7 |
| hemris plantar fascia bone erosion right^a^ | 67 | 13 | 16.2 |
| hemris plantar fascia bone marrow oedema right^a^ | 62 | 18 | 22.5 |
| hemris plantar fascia intraaponeurosis hypersignal right^a^ | 68 | 12 | 15 |
| petct synovitis shoulder right^b^ | 75 | 5 | 6.3 |
| petct synovitis shoulder left^b^ | 75 | 5 | 6.3 |
| petct synovitis elbow right^b^ | 76 | 4 | 5 |
| petct synovitis elbow left^b^ | 76 | 4 | 5 |
| petct synovitis carpus right^b^ | 74 | 6 | 7.5 |
| petct synovitis carpus left^b^ | 74 | 6 | 7.5 |
| petct synovitis hip right^b^ | 75 | 5 | 6.3 |
| petct synovitis hip left^b^ | 75 | 5 | 6.3 |
| petct synovitis knee right^b^ | 74 | 6 | 7.5 |
| petct synovitis knee left^b^ | 74 | 6 | 7.5 |
| petct synovitis ankle right^b^ | 76 | 4 | 5 |
| petct synovitis ankle left^b^ | 76 | 4 | 5 |
| shs lefthand erosions | 80 | 0 | 0 |
| shs righthand erosions | 80 | 0 | 0 |
| shs lefthand jsn | 80 | 0 | 0 |
| shs righthand jsn | 80 | 0 | 0 |
| shs left foot erosions | 78 | 2 | 2.5 |
| shs right foot erosions | 78 | 2 | 2.5 |
| shs left foot jsn | 78 | 2 | 2.5 |
| shs right foot jsn | 78 | 2 | 2.5 |
| meansuvmax ascending aorta^b^ | 71 | 9 | 11.3 |
| meansuvmax aortic arch^b^ | 73 | 7 | 8.8 |
| meansuvmax descending aorta^b^ | 76 | 4 | 5 |
| meansuvmax suprarenal aorta^b^ | 75 | 5 | 6.3 |
| Meansuvmax infrarenal aorta^b^ | 74 | 6 | 7.5 |
| meansuvmean Superior Vena Cava (SVC)^b^ | 75 | 5 | 6.3 |
