# Supplementary material for: Multimodal imaging of structural damage and inflammation in psoriatic arthritis: a comparison of DMARD-naive and DMARD-failure patients
Source: Rheumatology (Oxford). 2024 Aug 17;64(4):1760–9. doi: 10.1093/rheumatology/keae450 (PMC11962931; doi:10.1093/rheumatology/keae450)

Supplementary Figure S1: Sub-scores of HEMRIS Structure Achilles Tendon for both patient groups. In tendon thickening (p=0.241), bone spur (p=0.094), and bone erosion (p=0.251) plots, each circle represents an individual and the line represents the median value of the group.


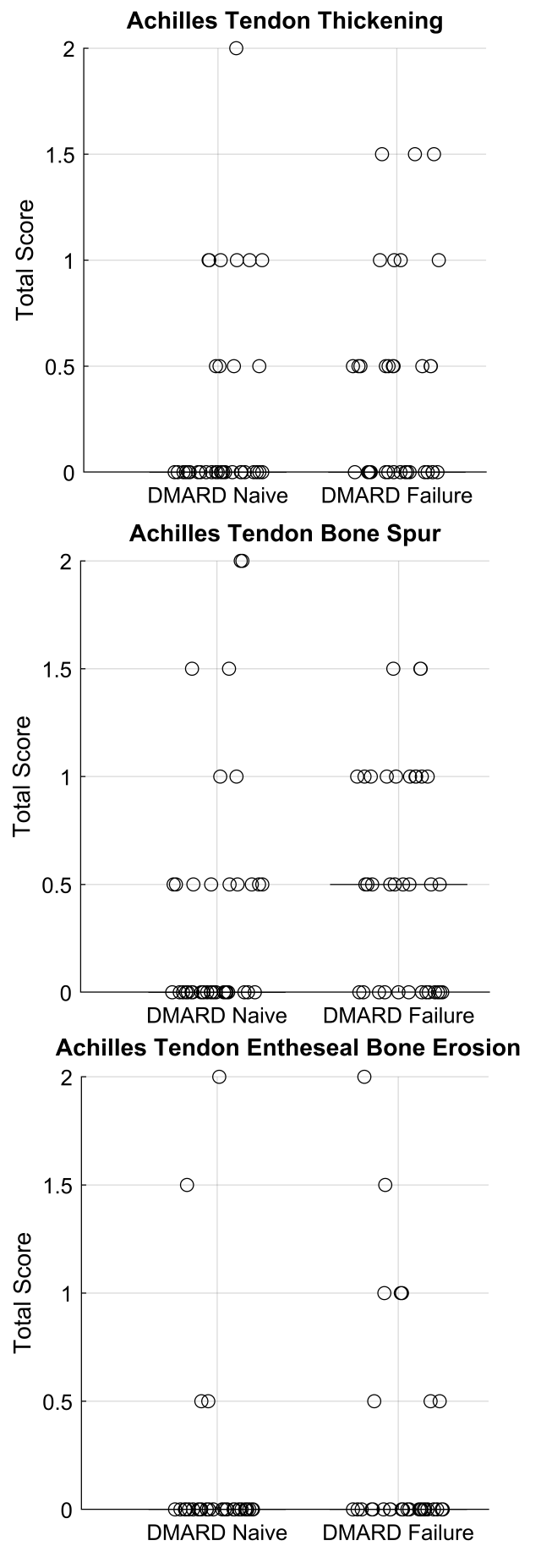


Supplementary Figure S2: Sub-scores of Sharp-van der Heijde (SHS) Joint Space Narrowing (JSN) for both patient groups. In JSN hands (p=0.201) and feet (p=0.022) plots, each circle represent an individual and the line represents the median value of the group.


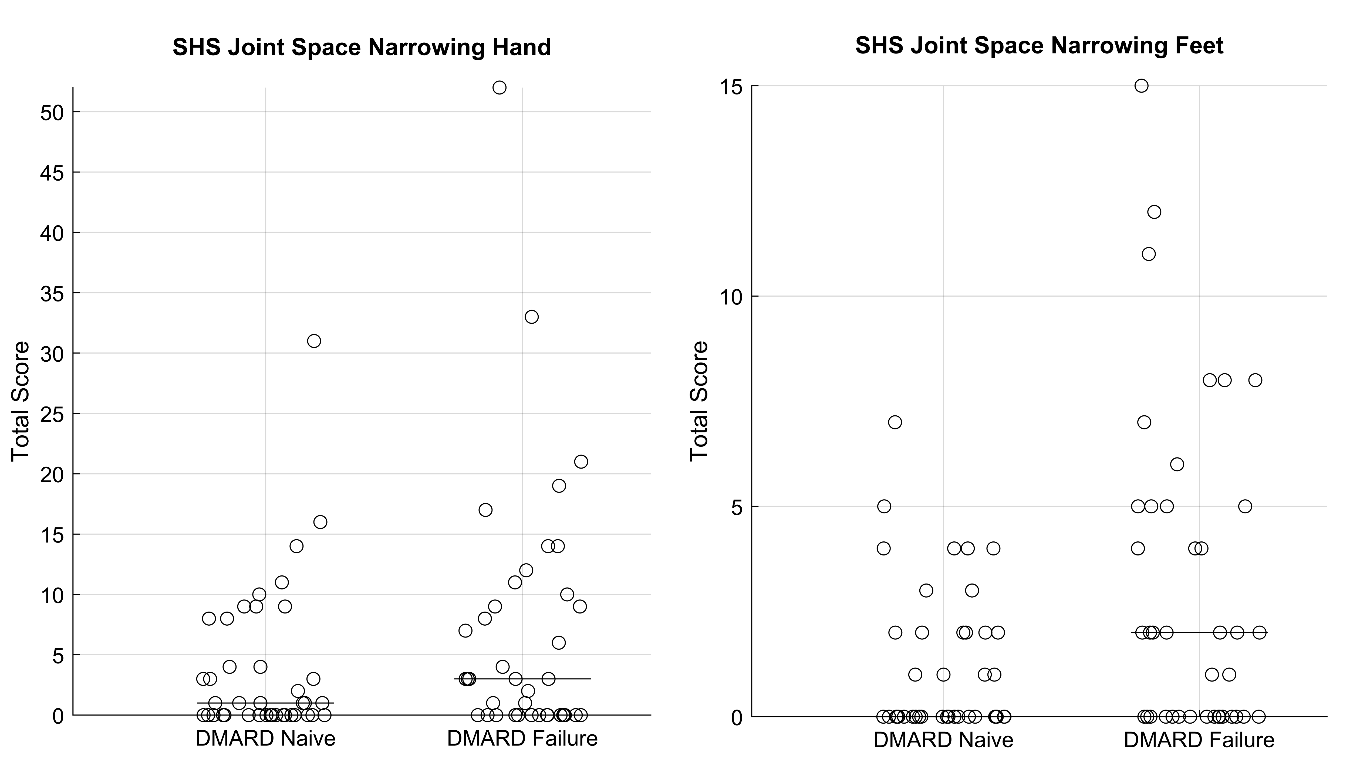


Supplementary Figure S3: Illustration of between group differences for inflammatory imaging parameters. Each circle represents an individual and gives the maximum value of right and left joints for that individual.


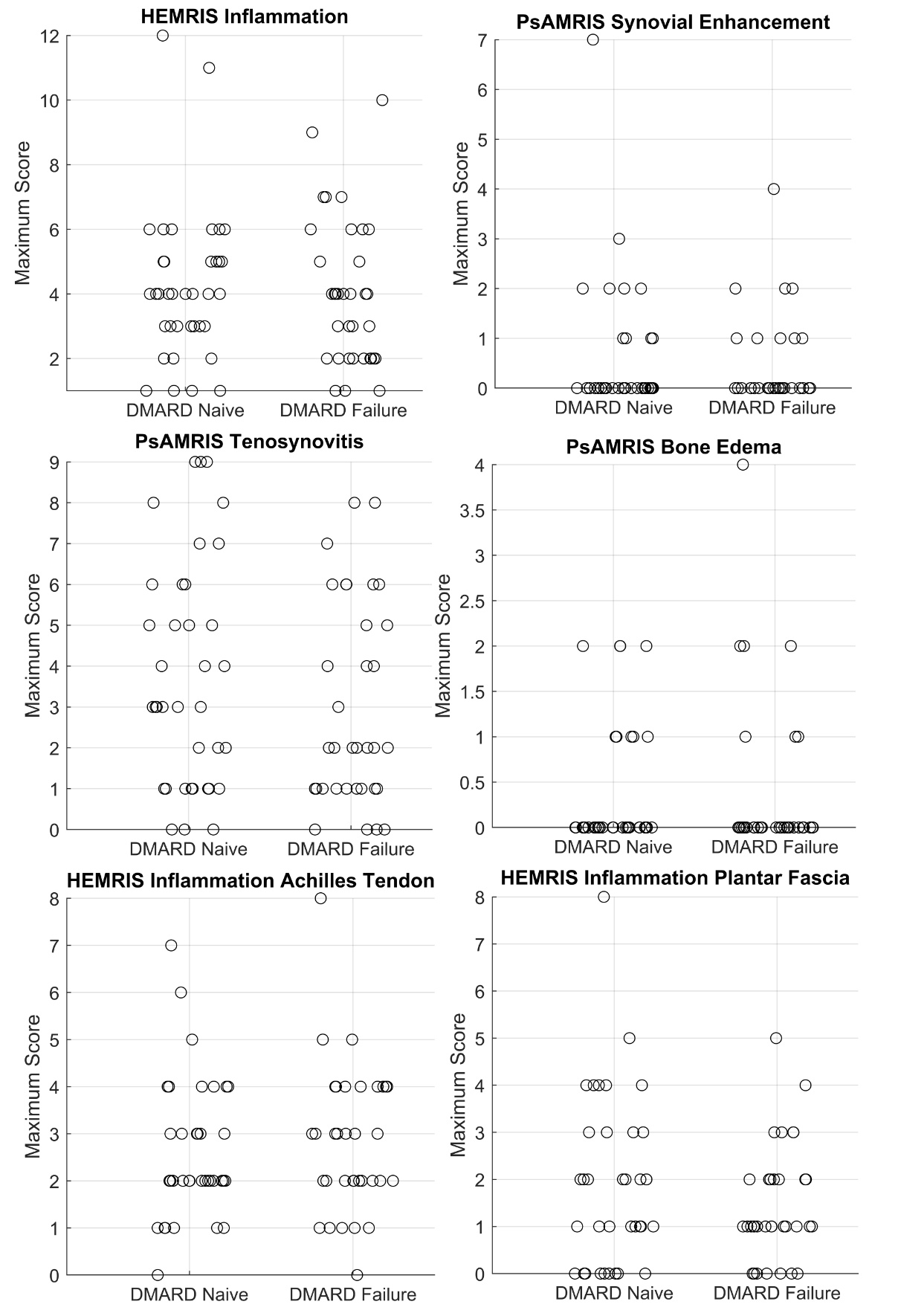


Supplementary Figure S4: Illustration of between group differences for structural imaging parameters. Each circle represents an individual and gives the maximum value of right and left joints for that individual.


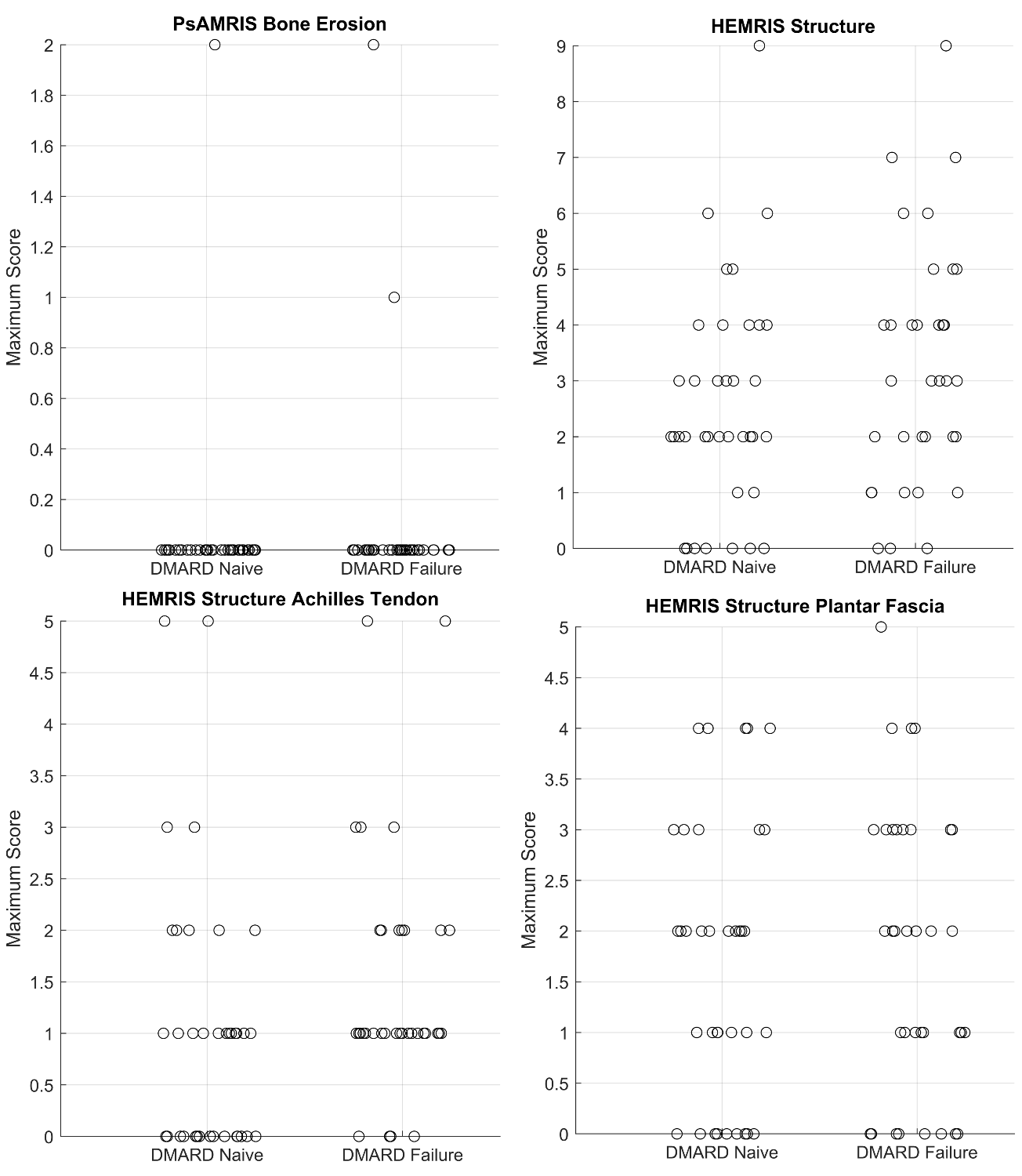

Supplement: keae450_Supplementary_Data [file keae450_supplementary_data.zip › keae450_Supplementary_Data/rhe-24-0820-File006.docx]
